# Seasonality influences key physiological components contributing to *Culex pipiens* vector competence

**DOI:** 10.3389/finsc.2023.1144072

**Published:** 2023-05-25

**Authors:** Eleanor N. Field, Ryan C. Smith

**Affiliations:** Department of Plant Pathology, Entomology and Microbiology, Iowa State University, Ames, IA, United States

**Keywords:** seasonality, *Culex pipiens*, mosquito, semi-field, physiology, *Wolbachia*, gene expression, RNAi

## Abstract

Mosquitoes are the most important animal vector of disease on the planet, transmitting a variety of pathogens of both medical and veterinary importance. Mosquito-borne diseases display distinct seasonal patterns driven by both environmental and biological variables. However, an important, yet unexplored component of these patterns is the potential for seasonal influences on mosquito physiology that may ultimately influence vector competence. To address this question, we selected *Culex pipiens*, a primary vector of the West Nile virus (WNV) in the temperate United States, to examine the seasonal impacts on mosquito physiology by examining known immune and bacterial components implicated in mosquito arbovirus infection. Semi-field experiments were performed under spring, summer, and late-summer conditions, corresponding to historically low-, medium-, and high-intensity periods of WNV transmission, respectively. Through these experiments, we observed differences in the expression of immune genes and RNA interference (RNAi) pathway components, as well as changes in the distribution and abundance of *Wolbachia* in the mosquitoes across seasonal cohorts. Together, these findings support the conclusion that seasonal changes significantly influence mosquito physiology and components of the mosquito microbiome, suggesting that seasonality may impact mosquito susceptibility to pathogen infection, which could account for the temporal patterns in mosquito-borne disease transmission.

## Introduction

Several mosquito-borne pathogens, such as malaria, dengue, and West Nile virus (WNV), display seasonal patterns in their transmission (1–6) that are driven by environmental variables that influence mosquito life-history traits (7–9), physiology (10, 11), and abundance (11–14), which ultimately shape mosquito-borne disease transmission (15). However, there is little information on how seasonal trends may influence mosquito susceptibility to pathogen infection.

The ability of a mosquito to transmit disease, otherwise known as vector competence, is a multivariate trait incorporating aspects of innate immunity, the microbiota, and host physiology (16–21). RNA interference (RNAi) pathways serve as the primary mechanism for controlling virus infection in the mosquito host (18–20). This includes the prominent roles of Dicer-2 (*DCR2*) and Argonaute-2 (*AGO2*) in the production of small interfering RNAs (siRNAs) that cause the targeted degradation of viral RNAs (18–20). In addition, the activation of the JAK-STAT, Toll, and IMD immune signaling pathways initiates the expression of downstream effector genes [such as cecropin (22, 23) and *Vago* (24, 25)] that limit virus infection (18, 19). The mosquito microbiome can also have a significant impact on vector competence, influencing infection outcomes for a variety of mosquito-borne pathogens (26–28).

The endosymbiont *Wolbachia pipientis* naturally infects a wide range of arthropods, including the mosquito *Culex pipiens* (29). Although *Wolbachia* is often associated with host alterations to reproduction (30), the presence of *Wolbachia* has been shown to influence mosquito arbovirus infection in both natural (31) and artificial hosts (32, 33). When paired with the previous observation that *Wolbachia*-induced arbovirus resistance is density dependent (32, 34), the presence of *Wolbachia* can have a significant impact on mosquito vector competence.

Temperature has been implicated as the primary driver of seasonal transmission dynamics (15, 35–41) and vector competence (37, 42), with known impacts on mosquito immune function (such as melanization and phagocytosis) (43, 44) and immune gene expression (43–45), which serve as the first line of defense against invading pathogens. Temperature has also been implicated in the shaping of the mosquito microbiota (46), most notably that of *Wolbachia* (47–49), which can have positive or negative impacts on arbovirus infection (32, 33). Moreover, temperature can also influence the efficacy of the RNAi pathway (50), which is integral to mosquito antiviral immunity (18–20). Although these studies demonstrate the complexity and widespread influence of temperature on the mosquito vector, it is currently unclear how temperature in the context of other seasonal factors, such as photoperiod and relative humidity, may together influence mosquito vector competence.

To approach this question, we performed semi-field experiments with a laboratory-derived strain of *Culex pipiens*, a primary vector of WNV in the United States, to examine potential seasonal differences in mosquito host physiology that could influence vector competence. We demonstrate that adult female *Cx. pipiens* reared under spring, summer, and late-summer conditions display distinct differences in the expression of immune genes and RNAi pathway components, and in *Wolbachia* abundance. These data suggest that mosquito physiology and vector competence is dynamic, where different seasonal environmental conditions may have a significant influence on mosquito susceptibility to pathogen infection. As a result, our data suggest that environmental conditions at different times of the year likely influence vector susceptibility and seasonal trends of mosquito-borne disease transmission that are potentially transferable to other vector–pathogen systems.

## Materials and methods

### *Culex pipiens* rearing and maintenance

A laboratory colony of *Cx. pipiens* mosquitoes, originally isolated from field collections in Ames, IA, United States, was maintained at 25°C, 85% relative humidity, and 16 : 8 hours (L : D; light : dark) on 10% sucrose *ad libitum*. Larvae were maintained using an equal mix of crushed Milk-Bone® and TetraMin® fish food, while commercial sheep’s blood (Hemostat Laboratories), provided via artificial membrane feeding, was used for blood feeding and subsequent egg production.

### Semi-field studies

**Figure 1:**
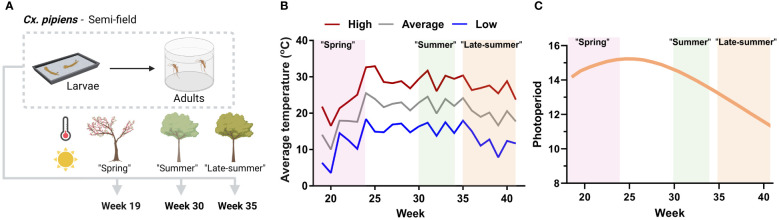
Overview and temperature conditions of the semi-field experiments examining *Culex pipiens* seasonality. **(A)** Graphical overview of semi-field experiments where first-instar *Culex pipiens* larvae were reared outside at three time points (week 19, 30, and 35 of the year) to represent spring, summer, and late-summer conditions. Mosquitoes were reared outside for their entire development, first in metal trays for larval growth, then the pupae were collected and placed in eclosion chambers to allow for adult emergence. Adult female mosquitoes were collected 5–8 days post emergence for further molecular analysis. Mosquitoes were obtained from a laboratory colony of *Cx. pipiens*. **(B)** Conditions experienced by each study group, with temperatures reflecting the weekly average of the daily high, average, and low temperatures for Ames, IA, United States, in 2021 when the experiments were performed. **(C)** Photoperiod (daylight) averages are displayed by week, highlighting conditions for each study group. Graphics in **(A)** were created with BioRender.com.

To determine how different seasonal environmental conditions might influence mosquito physiology, we performed semi-field experiments with a laboratory colony of *Cx. pipiens*, in a similar manner to previous studies (11) at different times of the year. Initiated with first-instar larvae, mosquitoes were reared to adulthood under spring (week 19, May), summer (week 30, July), and late-summer (week 35, late August) conditions (**Figure 1A**). These time points are representative of periods of little-to-no WNV activity, increasing WNV activity, and peak WNV activity, respectively, which ultimately shape the seasonality of WNV transmission (5, 6).

For the semi-field studies, first-instar larvae from our laboratory colony of *Cx. pipiens* were placed in metal rearing trays covered by a tempered glass pane to provide protection from the elements, contaminants, or potential predation. Rearing trays were placed at two locations in Ames, IA, United States, at epidemiological weeks 19 (9 May), 30 (25 July), and 35 (29 August) in 2021, coinciding with periods of differing mosquito activity and WNV transmission intensity in the state of Iowa (5, 11, 14, 51, 52). The use of adult mosquitoes at week 30 corresponded with previous semi-field studies (11), with the resulting mosquito samples shared between experiments. Larval density in each experimental condition was approximately 300 per tray in 1 L of distilled water, with larvae maintained on an equal mix of crushed Milk-Bone® and TetraMin® fish food. Resulting pupae were collected and placed in special eclosion chambers (BioQuip) directly adjacent to the larval rearing trays where, following emergence, adults were maintained on 10% sucrose under semi-field conditions until the collection of mosquito samples and sheltered under a metal cage to provide protection from animal disturbance. The last cup of adults was collected on 15 October (week 41), terminating the semi-field study.

### Environmental data

Daily high and low temperatures (°C) were collected for the study period from May to October using the southeast Ames station (IA0203) of the Iowa State University Mesonet (https://mesonet.agron.iastate.edu) to understand semi-field temperature conditions. A 30-year average was provided from the central Iowa station (IA50014) to place the study year’s values in a larger context. Diurnal temperature ranges were determined using the central Iowa station as the difference between the average daily high temperature and average daily low temperature for each week. Photoperiod data were obtained from an online sunrise/sunset table for Des Moines, IA, United States (www.timeanddate.com), with hours of daylight displayed as average weekly values. Data were visualized in R (version 3.6.3) using the ggplot2 package.

### Gene expression analysis

Adult female *Cx. pipiens* (5–8 days post eclosion) were collected from laboratory or semi-field samples and stored at –80°C for later processing. Mosquitoes were pooled into groups of 5–10 mosquitoes for analysis, with three or more replicates per experimental treatment. RNA was extracted from stored samples using Trizol® and cDNA was synthesized using the RevertAid cDNA kit (Thermo Fisher Scientific). Immune genes previously implicated in antiviral immunity were selected from previous studies (24, 50), with primer sequences listed in **Table S1**. Relative gene expression was measured via quantitative reverse transcription-polymerase chain reaction (qRT-PCR) using a Quant Studio 3 (Thermo Fisher Scientific) and PowerUp SYBR Green (Thermo Fisher Scientific) under the following conditions: 50°C for 2 min, 95°C for 15 s, and 60°C for 1 min for 40 cycles.

### *Wolbachia* quantification in *Culex pipiens*

An initial PCR was performed to amplify an approximately 900-bp fragment of the *Wolbachia* 16S ribosomal RNA gene from our laboratory colony of *Cx. pipiens* using previously described methods with *Wolbachia*-specific primers (53) (**Table S2**). Following cloning and Sanger sequencing, the resulting 16S sequence was deposited in GenBank (accession number OQ034610). From this sequence, specific *Wolbachia* primers derived from our *Cx. pipiens* colony (ISU Wolb), amplifying a 249-bp amplicon, were designed to determine relative *Wolbachia* titers when normalized to transcript levels of ribosomal protein L32 (*Rpl32*) (48, 54) (**Table S2**). To determine *Wolbachia* titers across laboratory and seasonal conditions, dissected ovaries and ovariectomized carcasses were stored at –80°C until further processing. DNA was isolated using the Marriot DNA extraction procedure as described previously (5, 55–57) and stored at –20°C prior to future use. Amplification was performed by qRT-PCR using a Quant Studio 3 (Thermo Fisher) and PowerUp SYBR Green (Thermo Fisher) under the following conditions: 50°C for 2 min, 95°C for 15 s, and 60°C for 1 min for 40 cycles. *Wolbachia* titers were determined by calculating the delta Ct of *Wolbachia* copies relative to *Rpl32* and transformed (2^n^) to produce relative *Wolbachia* density estimates for each tissue sample (48, 54).

### Statistical analyses

All statistical analyses were performed using GraphPad (Prism version 7). One-way non-parametric ANOVA tests (Kruskal–Wallis) with Dunn’s *post hoc* analysis being used to test differences in gene expression and *Wolbachia* titers.

## Results

### Semi-field studies to address mosquito seasonality

To assess the seasonal conditions of our semi-field studies (outlined in **Figure 1A**), we examined the effects of temperature (**Figure 1B**) and photoperiod (**Figure 1C**) for each of the semi-field cohorts during our study period.

The spring cohort experienced the largest variation in temperature, recording the coldest weekly low temperature of 3.6°C in week 20 and the warmest weekly high of 32.6°C in week 24 of our study period (**Figure 1B**, **Table 1**). In contrast, the summer cohort experienced the most stable temperature conditions, with little fluctuation in daily temperature ranges, compared with the other experimental groups (**Figures 1**, **S1**). The late-summer cohort faced declining temperatures during its development, with daily lows dipping below 10°C in week 39 (**Figure 1B**). Although life-history traits were not directly collected in these experiments, the summer (week 30) cohort had the shortest developmental time and highest percentage of larvae reaching adulthood. By comparison, mosquito development in both the spring and late-summer cohorts was extended. Together, these data suggest that weekly temperature fluctuations are much more dynamic in the spring and late summer, such that the effects of temperature may influence mosquito development and physiology very differently than the more uniform summer conditions.

**Table 1:** Seasonal conditions of each semi-field cohort.

Group	Weeks active^§^	Average high (°C)	Average (°C)	Average low (°C)
Spring	19–24	23.4	17.2	10.9
Summer	30–34	29.4	22.7	15.9
Late summer	35–41	27.0	19.9	12.7

**^§^** Defined as the weeks from when the first-instar larvae were placed in semi-field conditions to when adults were collected for processing.

An additional measurable aspect of our seasonal conditions is the photoperiod, for which the spring cohort experienced the highest levels, averaging 14 hours and 46 minutes of daylight (**Figure 1C**). The amount of daylight decreased across the other seasonal cohorts, with the summer condition experiencing a slight decrease (14 hours and 12 minutes) and the late-summer cohort subjected to a prominent decrease in photoperiod of approximately 2 hours (12 hours and 23 minutes; **Figure 1C**).

### Seasonality influences key components of mosquito antiviral immunity

**Figure 2:**
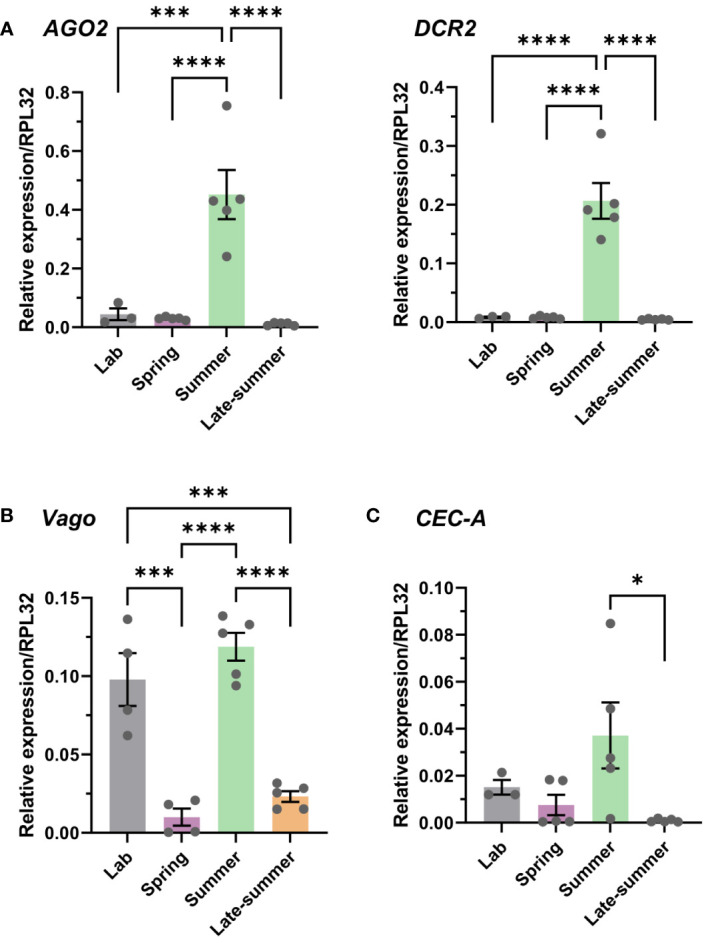
Seasonality of *Culex pipiens* immune gene expression. Gene expression was examined using samples from our *Cx. pipiens* colony under standard rearing conditions (laboratory) and across seasonal conditions (spring, summer, and late summer) from semi-field experiments. Expression of **(A)** genes involved in the RNA interference (RNAi) pathway (*AGO2* and *DCR2*), **(B)** the immune factor Vago, or **(C)** the antimicrobial gene *CEC-A* were evaluated by quantitative reverse transcription- polymerase chain reaction (qRT-PCR). Each replicate contained groups of four pooled mosquitoes, with three or more independent samples per experimental condition. Data are displayed as the mean ± SEM, with significance determined using a one-way ANOVA with a Tukey’s multiple comparison test. Significant differences between conditions are denoted by asterisks (*, *p* < 0.05; ***, *p* < 0.001; ****, *p* < 0.0001); all other comparisons between conditions were not significant. SEM, standard error of the mean.

With previous studies demonstrating the effects of temperature on mosquito immune function (43–45, 50), we examined immune gene expression in the context of our seasonal conditions. With RNA interference (RNAi) considered the primary mechanism of antiviral immunity in mosquitoes (58, 59), we determined the relative gene expression of two primary RNAi pathway components, Dicer-2 (*DCR2*) and Argonaute-2 (*AGO2*) (50, 58) in the context of our semi-field experiments. Both genes displayed significantly increased expression during the summer cohort when compared with the laboratory colony or other experimental semi-field groups (**Figure 2A**), suggesting elevated RNAi activity and increased antiviral defenses during this time period.

In addition, we also examined *Vago* and cecropin A (*CEC-A*) expression, immune genes previously implicated in the *Culex* antiviral response to WNV (24, 25) and as an important antimicrobial peptide acting on a wide range of pathogens (60, 61) including viruses, respectively (22, 23). *Vago* displayed reduced levels of expression during the spring and late-summer conditions (**Figure 2B**), whereas *CEC-A* displayed a similar, less pronounced phenotype with significantly reduced expression during the late-summer time period (**Figure 2C**). This suggests that *Vago-* and *CEC-A*-mediated immune responses may be attenuated during these seasonal time points. Together, these data suggest that important mediators of the mosquito immune response and antiviral immunity are influenced by seasonality, suggesting that mosquito susceptibility to pathogen infection may be highly variable during the course of the season.

### Seasonality influences *Wolbachia* abundance and tissue localization

**Figure 3:**
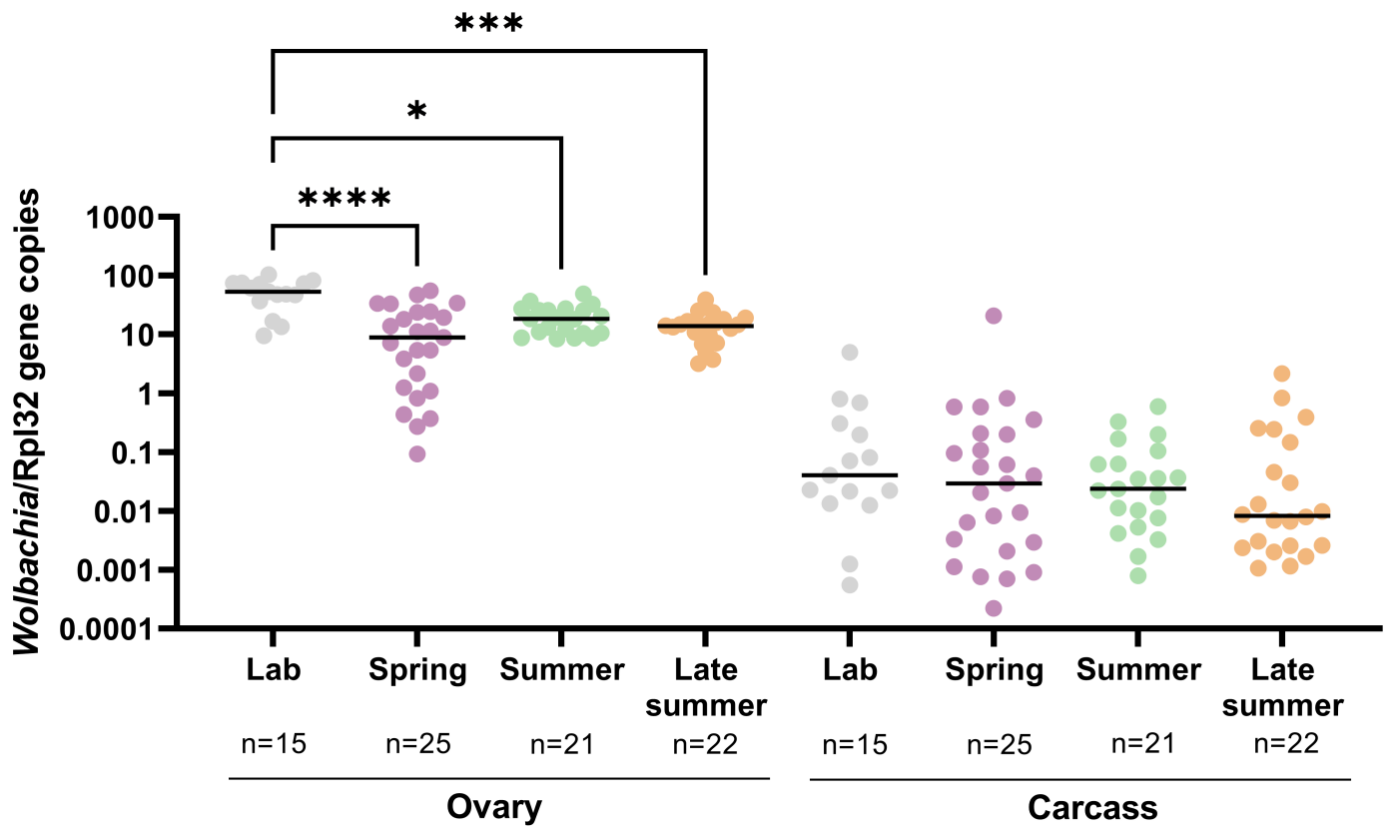
Seasonality of *Wolbachia* titers in *Culex pipiens* ovary and carcass samples. *Wolbachia* titers (16s RNA) were examined in individual *Cx. pipiens* ovary or carcass (remaining tissue following ovary dissection) samples reared in the laboratory or under spring, summer, and late-summer semi-field conditions by quantitative reverse transcription- polymerase chain reaction (qRT-PCR) and normalized to *Rpl32* expression. A one-way non-parametric ANOVA (Kruskal–Wallis) with a Dunn’s multiple comparison test was used to determine significance. Significant differences between conditions are denoted by asterisks (*, *p* < 0.05; ***, *p* < 0.001; ****, *p* < 0.0001).

*Cx. pipiens* are natural hosts of *Wolbachia*, insect endosymbionts that influence mosquito vector competence for arbovirus infection (32, 33). Although *Wolbachia* dynamics in response to temperature have been explored extensively in other mosquito species (47–49), the impacts of temperature on *Wolbachia* in *Cx. pipiens* have not been previously explored. Although *Wolbachia* are commonly associated with germ-line tissues, *Wolbachia* can also be found within somatic tissues of *Culex* species (33, 54, 62). For this reason, we specifically examined *Wolbachia* titers by qRT-PCR (54) in the ovaries and the dissected ovariectomized carcass of adult females reared in the laboratory and our semi-field conditions (**Figure 3**). When compared with the results from laboratory-reared mosquitoes, the *Wolbachia* titers in the ovaries of mosquitos reared in semi-field conditions were significantly reduced across seasonal conditions, with the reduction in *Wolbachia* the most prominent at the spring time point (**Figure 3**), thus demonstrating that *Wolbachia* abundance in the mosquito ovary notably changes throughout the year. As expected, *Wolbachia* abundance was substantially higher in germline tissue than in somatic tissue (**Figure 3**). *Wolbachia* levels were slightly reduced in somatic tissues during the late summer, yet did not display similar differences across seasonal time points (**Figure 3**).

## Discussion

Although the seasonal trends of mosquito-borne diseases are well established, few studies have attempted to understand the mechanisms by which mosquito vector competence may change across seasons. To begin to address this question, we performed semi-field studies to measure changes in components that influence mosquito vector competence (immune gene expression and *Wolbachia* dynamics) in *Cx. pipiens* across seasonal time points that reflect periods of low- to high-WNV transmission trends.

WNV exhibits clear seasonal transmission trends in *Culex* populations and human cases, with infections peaking in the late summer across the United States (5, 6), yet the mechanisms driving these seasonal trends have been largely unexplored. Although previous studies have suggested that the spatiotemporal abundance of *Culex* vector populations in the summer and late summer can lead to increased levels of WNV infection in mosquito populations (14, 52), overall *Culex* abundance in Iowa typically peaks much earlier in the season (i.e., May/June) (5, 11, 14, 51, 52). Therefore, combining both mosquito abundance and infection data (vector index) is more predictive than mosquito abundance alone in predicting human WNV cases (63–66), demonstrating the importance of identifying the physiological components that contribute to mosquito infection and transmission of WNV. Although climate conditions are known to influence interannual differences in mosquito populations (14) and promote virus amplification (35, 41), the influence of seasonal changes on mosquito physiology and vector competence have not been adequately addressed outside of the context of diapause (11, 67, 68). Through the use of seasonal cohorts in our semi-field studies, we have demonstrated that components of the RNAi pathway and immune-related genes are differentially expressed over the spring, summer, and late-summer seasons, suggesting that the susceptibility of *Cx. pipiens* to infection is highly variable throughout the year. In addition, differences in *Wolbachia* abundance and localization in somatic and germline tissues may similarly contribute to *Cx. pipiens* physiology and vector competence.

Temperature, a key component of seasonality, has previously been found to have an effect on mosquito immune function (43) and vector competence in virus infection (38–40). In *Culex tarsalis*, increasing temperatures resulted in fewer infected females following exposure to WEE (69), suggesting that the antiviral immune response was stronger at higher temperatures. This is further supported by the results from a study by Adelman et al. (50), which used a transgenic *Aedes aegypti* sensor strain to investigate the impacts of temperature on the RNAi pathway, demonstrating the destabilization of RNAi at cooler temperatures (18°C). Our results show that the summer-reared group (experiencing uniformly warm temperatures) displayed the highest expression of the RNAi machinery components *Dcr-2* and *Ago-2*. These genes were expressed at much lower levels during the spring and late summer when mosquitoes experienced cooler temperatures. This indicates that the RNAi pathway may be attenuated during these seasonal time frames, corresponding with the initiation of viral cycling in the spring and increased frequency of mosquito WNV infections in the late summer. However, these observations should be directly tested in the future through WNV infection experiments under seasonal conditions, and, as a result, the lack of direct testing represents a limitation in the interpretation of our current study.

Similar trends were also found for other components of the immune system, such as *Vago* and CEC-A, which also displayed reduced levels of expression in the spring and late summer. *Vago* has been described as an interferon-like cytokine that activates the Jak-STAT pathway to limit WNV infection in *Culex quinquefasciatus* (24), whereas cecropins and other antimicrobial peptides (AMPs) have been implicated in antiviral immunity in insects (22, 23). Although the functional role of *Vago* has not previously been explored in the context of seasonality, previous studies have demonstrated that cecropin expression is influenced by temperature (42, 43). Together, our data suggest that the downregulation of these genes during the spring and late summer likely contribute to seasonal differences in *Culex* vector competence and susceptibility to WNV infection, although these conclusions need to be further tested in future WNV infection experiments.

In addition to components of the innate immune system, microbes can also modulate mosquito physiology and vector competence in important ways. *Wolbachia* are insect endosymbionts that affect many aspects of host biology, from reproduction (30, 70) to pathogen infection (32, 33, 71, 72). *Wolbachia* are distinct from the general gut microbiota, as they are intracellular bacteria distributed in both somatic and germ-line tissues (62). Although our data suggest that changes in ovary *Wolbachia* titers could influence cytoplasmic incompatibility and reproduction, we did not directly address whether or not these changes have measurable reproductive phenotypes and will look to address these questions in future experiments. Similar to previous studies evaluating *Wolbachia* in field-collected *Cx. pipiens* (54), we found that somatic titers of *Wolbachia* were highly variable in laboratory samples and in samples from each of our semi-field conditions. Considering previous evidence that indicates that somatic infection densities of *Wolbachia* may contribute to WNV resistance (54), it is possible that the decrease in somatic *Wolbachia* levels in the late summer could influence *Cx. pipiens* vector competence and susceptibility to WNV infection. However, the lack of significant differences in somatic *Wolbachia* titers across our seasonal conditions suggests that any alterations to vector competence result from the highly variable somatic *Wolbachia* infections between individual mosquito samples as opposed to seasonal conditions.

Diurnal temperature fluctuations (i.e., the change between day and night temperatures) are most pronounced during the spring and late summer in temperate climates, and have previously been suggested to influence mosquito vector competence (73–77). This includes recent studies where diurnal temperature fluctuations influenced WNV titers in both *Cx. tarsalis* and *Cx. quinquefasciatus*; however, these differences in viral titers did not significantly influence infection, dissemination, or transmission (76). However, these data suggest that the variations in diurnal temperature correspond with seasonal changes, which may have additional impacts on mosquito host physiology and antiviral immunity. As a result, the seasonal differences in immune gene expression and *Wolbachia* abundance identified in our study may account for these physiological changes, thereby warranting further laboratory studies to distinguish the effects of seasonal temperature differences and diurnal temperature fluctuations.

Additional seasonal influences beyond that of temperature, such as changes in photoperiod and humidity, have been less explored in the context of mosquito vector competence. Although the combined effects of photoperiod and temperature are essential for promoting mosquito diapause (11, 78), the evidence suggests that photoperiod alone can influence mosquito size, lifespan, and propensity for blood-feeding (79). This suggests that other aspects of mosquito physiology may be influenced by photoperiod, yet these have not been adequately explored to date. Although humidity is also an important determinant of mosquito lifespan, recent evidence indicates that mosquito dehydration can influence carbohydrate metabolism and blood-feeding behavior, with the potential to increase pathogen transmission (80). However, future studies are needed to fully determine the impacts of humidity and dehydration on mosquito vector competence.

Although these experiments were conducted in mosquitoes reared under semi-field conditions, we believe that our results can similarly be used to interpret trends in natural populations of *Cx. pipiens*. Our seasonal observations of elevated immune expression in the summer, contrasted by impaired immune function in the spring and late summer, closely correspond to previous studies demonstrating seasonal differences in the vector competence of *Cx. tarsalis* populations collected from the field (81). Reisen et al. show that higher levels of western equine encephalitis virus were required to infect mosquitoes during the summer months, whereas mosquitoes were much more susceptible to virus infection in the spring and late-summer/fall months, demonstrating important seasonal differences in mosquito vector competence (81). As *Cx. tarsalis* is not naturally infected with *Wolbachia* such as *Cx. pipiens* (33), it is possible that changes to the efficiency of RNAi or immune expression, similar to those in our own results, could account for these observations of seasonal differences in infection outcomes.

Although further studies are required to fully delineate the seasonal influences on mosquito physiology and their impacts on mosquito-borne pathogen infection, we believe that our experiments are an important first step in evaluating the seasonal influences that determine mosquito vector competence.

## Data availability statement

The datasets presented in this study can be found in online repositories. The names of the repository/repositories and accession number(s) can be found in the article/**Supplementary Material**.

## Author contributions

EF and RS designed the study. EF performed experiments. EF and RS performed the analysis and wrote the manuscript. Both authors contributed to the article and approved the submitted version.
