# Supplementary material for: Seasonality influences key physiological components contributing to Culex pipiens vector competence
Source: Front Insect Sci. 2023 May 25;3:1144072. doi: 10.3389/finsc.2023.1144072 (PMC10926469; doi:10.3389/finsc.2023.1144072)
Supplement: Supplementary file 1 [file DataSheet_1.pdf]

Supplemental Information

Seasonality influences key physiological components contributing to *Culex pipiens* vector competence

Eleanor N. Field and Ryan C. Smith*

Department of Plant Pathology, Entomology and Microbiology, Iowa State University, Ames, Iowa 50011

*Corresponding author: smithr@iastate.edu

Supplemental Information

Supplemental Figures

Figure S1. Diurnal temperature range during the semi-field study.

Supplemental Tables

Table S1. Primer sequences used to measure for gene expression.

Table S2. Primer sequences used for *Wolbachia* quantification.

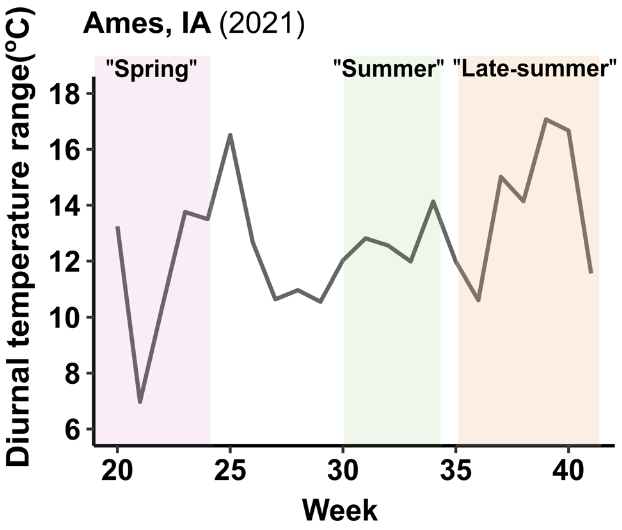

Figure S1. Diurnal temperature range during the semi-field study. Temperature differences between the weekly average high and low temperatures (°C) are displayed as the diurnal temperature range at the field location in Ames, IA (2021). The timing for which spring, summer, and late-summer field cohorts were deployed are denoted respectively by color shading. The weeks of the spring and late-summer-reared groups experienced the most fluctuations in daily temperature ranges.

Table S1. Primer sequences used to measure for gene expression.

Gene	Acc. No.	Forward	Reverse
**RPL32**	CPIJ001220	AAGCCGAAAGGTATCGACAA	CAGTAGACGCGGTTCTGCAT
**Vago**	CPIJ000699	TGAAGACATTCGGTGTGATCATCT	CAAGTTGTGTAGGTCAGCGAGAGA
**CecA**	CPIJ010699	AAACAGTCCCAAGGGAAAGTCAAC	TCCAACTTCTTGCCAAACTTCTTC
**Dcr-2**	CPIJ010534	GATGAAAATTCACACCTTCGATCC	GCAATGAAGTCCTCGTATTTGTCC
**Ago2**	CPIJ009898	GTTCTCGATGCAGCAGACGTACTA	CCTTGATACACTGCGTCAACAGAC

Table S2. Primer sequences used for *Wolbachia* identification and quantification.

Gene	Forward	Reverse
**Wolbachia 16S**	TTGTAGCCTGCTATGGTATAACT	GAATAGGTATGATTTTCATGT
**RPL32**	AAGCCGAAAGGTATCGACAA	CAGTAGACGCGGTTCTGCAT
**ISU Wolb**	TCTGGTTCAAATCTGACGCTGAAG	GAAGTGGTAAGGTTTTTCGCGTTG
